# Determinants of defaulting from completion of child immunization in Laelay Adiabo District, Tigray Region, Northern Ethiopia: A case-control study

**DOI:** 10.1371/journal.pone.0185533

**Published:** 2017-09-27

**Authors:** Hailay Gebretnsae Aregawi, Tesfay Gebregzabher Gebrehiwot, Yamane Gebremariam Abebe, Kidanu Gebremariam Meles, Alem Desta Wuneh

**Affiliations:** 1 Tigray Health Research Institute, Tigray Regional Health Bureau, Mekelle, Ethiopia; 2 School of Public Health, College of Health Sciences, Mekelle University, Mekelle, Ethiopia; University of Washington, UNITED STATES

## Abstract

**Background:**

Globally 2.5 million children under five years of age die every year due to vaccine preventable diseases. In Tigray Region in Northern Ethiopia, full vaccination coverage in children is low. However, the determinants of defaulting from completion of immunization have not been studied in depth. This study aimed to identify the determinants of defaulting from child immunization completion among children aged 9–23 months in the Laelay Adiabo District, North Ethiopia.

**Methods:**

An unmatched community based case-control study design was conducted among children aged 9–23 months in the Laelay Adiabo District from February—March 2015. A survey was conducted to identify the existence of cases and controls. Two hundred and seventy children aged 9–23 months (90 cases and 180 controls) were recruited from 11 kebeles (the smallest administrative units) by a simple random sampling technique using computer based Open Epi software. Cases were children aged 9–23 months who missed at least one dose of the recommended vaccine. Controls were children aged 9–23 months who had received all recommended vaccines. Data were collected from mothers/care givers using structured pretested questionnaire. The data were entered into Epi Info version 3.5.1 and analyzed using Statistical Package for Social Sciences (SPSS) version 21. Bivariate and Multiple logistic regression analysis were used to identify the predictors of the outcome variable. The degree of association was assessed by using odds ratio with 95% Confidence Interval (CI).

**Result:**

This study shows that mothers who take >30 minutes to reach the vaccination site (Adjusted Odds Ratio (AOR) = 3.56,95%CI:1.58–8.01); households not visited by health extension workers at least monthly (AOR = 2.68,95%CI:1.30–5.51); poor participation in women's developmental groups (AOR = 3.3,95%CI 1.54–7.08); no postnatal care follow-up (AOR = 5.2,95%CI:2.36–11.46); and poor knowledge of child immunization (AOR = 3.3,95%CI:1.87–7.43) were predictors of defaulting from completion of child immunization.

**Conclusion:**

Postnatal care follow-up, household visits by health extension workers and maternal participation in women’s development groups are important mediums for disseminating information and increasing knowledge to mothers about child immunization. To reduce the rate of defaulters, health providers should motivate and counsel mothers to attend postnatal care. Health extension workers should visit households at least once per month and strengthen mothers’ participation in the women’s development groups.

## Introduction

Immunization is one of the most efficient and successful health interventions for the reduction of child morbidity and mortality [1]. The term “defaulter” refers to children who miss scheduled vaccinations for any reason, including health facility problems such as canceled sessions or vaccine stock outs [2]. The World Health Organization (WHO) established the Expanded Program on Immunization (EPI) in 1974 to ensure universal access to routinely recommended childhood immunizations [3, 4].

Globally, an estimated 2.5 million under-five children die every year due to vaccine preventable diseases [1]. In 2013, coverage of third-dose diphtheria, tetanus, and pertussis vaccine (DTP3) among children aged < 12 months was 84% globally, ranging from 75% in the African Region to 96% in the Western Pacific and European Regions. About 14.8 million (68%) children who did not receive the DTP3 vaccine during the first year of life lived in 10 countries (India, Nigeria, Pakistan, Ethiopia, Democratic Republic of Congo, Indonesia, Vietnam, Mexico, South Africa and Kenya) [5]. In 2007, over 10% of children under one year in low-income countries were not receiving even the first dose of DTP, compared with just 2% in industrialized countries. Most under-immunized children live in the low-income countries that are also challenged by insufficient health service infrastructure, difficult topography, and armed conflict [6].

EPI was rolled out in Ethiopia in 1980 with the aim of reducing child mortality and morbidity [3, 7, 8]. Initially, only six vaccine preventable diseases were included in Ethiopia’s routine immunization program for under-five children: tuberculosis, poliomyelitis, tetanus, diphtheria, pertussis, and measles [9]. The currently routine immunization service includes ten vaccines preventable diseases: measles, diphtheria, haemophilus influenza type B, tetanus, pertussis, hepatitis B, pneumococcal disease, poliomyelitis, rotavirus infections and tuberculosis [7]. The National Immunization Schedule for Infants in Ethiopia is shown in **Table 1**.

**Table 1:** National immunization schedules for infants in Ethiopia.

Type of vaccine	Age (When to give)
BCG, OPV0	At birth
(DPT-HepB-Hib)1,OPV1,PCV1, Rota1	6 weeks
(DPT-HepB-Hib)2,OPV2,PCV, Rota2	10 weeks
(DPT-HepB-Hib)3, OPV3, PCV3	14 weeks
Measles	9 months

BCG—Bacillus Calmette-Guerin; OPV—Oral Polio Vaccine; DPT–HepB—Hib—Diphtheria, Pertussis, Tetanus, Hepatitis B, Haemophilus Influenza Type B; PCV—Pneumococcal Conjugate Vaccine; Rota—Rotavirus Vaccine.

Based on an Ethiopian Federal Ministry of Health report in 2013, measles and full vaccination coverage were 83.2%, and 77.7% [10]. However, an independent research study from 2012 shows that full vaccination coverage in Ethiopia was only 50%, and from those, only 18.6% received the immunizations on schedule and with appropriate space between doses. The study found that full vaccination was higher among children of more educated mothers/caregivers, children of first parity, and those residing in urban areas [11].

Many peer-reviewed studies have reported that socio-economic and demographic characteristics, access to health services and maternal knowledge about immunizations are factors that affect the completion of childhood immunization [12–17]. However, these studies were cross-sectional and also failed to include certain variables or factors that could be pertinent in the Ethiopian context, such as maternal participation in grassroots community groups/women’s development groups.

In Tigray Region, the main barrier to child immunization is not the first dose; rather, timeliness and appropriate spacing between doses are the main challenges. In 2012 in Tigray Region, full vaccination coverage was 77.9%, however, only 36.3% of children were completing immunizations on schedule and with appropriate spacing between doses [11]. There is no recently published independent research on child immunization in Tigray Region. Therefore, this study aimed to identify the determinants of defaulting from completion of child immunization. The results can be used by health providers and administrators to improve the coverage of full immunization in children, and additionally by policy makers and program managers to formulate appropriate strategies to decrease the “defaulter” rate.

## Methods

### Study design and area

An unmatched community based case-control study design was conducted to assess the determinants of defaulting from completion of immunization among children aged 9–23 months in the Laelay Adiabo District from February-March 2015. Laelay Adiabo District is 1,116 kilometers from Addis Ababa and 335 kilometers from Mekelle, the capital of Tigray Region. There are 22 kebeles (the smallest administrative units) in the district. According to the 2007 Ethiopian Census projections, Laelay Adiabo District had 19,944 under-five children and 3,978 infants in 2015. There are 127 health professionals and 51 health extension workers (HEWs) in the district with one primary hospital, four health centers and 17 health posts supported by 39 outreach vaccination sites which routinely provide immunization services.

### Source population and study population

The source population was all children between ages of 9–23 months who had started at least one dose of the routine immunization program in the Laelay Adiabo District. The study population was all children residing in 11 randomly selected kebeles. Cases were children (9–23 months) who had missed at least one dose of the recommended vaccine schedule. Controls were children between ages of 9–23 months who had completed the entire recommended vaccine schedule.

### Sample size determination

We computed the sample size by considering the predictor variables from previous case-control studies conducted in Ethiopia [18, 19]. Maternal knowledge of the vaccination schedule was selected since it gave the maximum sample size. Among controls, 9.4% of mothers lacked knowledge of the vaccination schedule, while among cases it was 23.6% (18). Using the assumptions of 80% power, 95% confidence interval, 10% non-response rate and a case-to-control ratio of 1:2, the total sample size was 270 (90 cases and 180 controls) (**Table 2**).

**Table 2:** Sample size calculation by using predictors variables from previous study.

Significant predictors	Reference	CI(%)	Power	Case:	% of exposure		OR	Samples size including 10%		
			(%)	Control	Case	Control		Case	Control	Total
1.Lack of maternal Knowledge on schedule of vaccines	**-18**	**95**	**80**	**1:02**	**23.60%**	**9.40%**	**3**	**90**	**180**	**270**
2.Monthly family income (having monthly income less 22USD)	-18	95	80	1:02	89.30%	72.70%	2.3	73	146	222
3.Postponed vaccines Schedule	-18	95	80	1:02	83.30%	4.50%	2	10	20	30
4.PNC visit (no visit)	-18	95	80	1:02	40.10%	11.10%	20	30	60	90
5.Maternal educational level (illiterate)	-19	95	80	1:02	71.80%	31.20%	3.6	20	40	60
6.Place of delivery (at home)	-19	95	80	1:02	90.30%	61.00%	4.1	19	38	57
7.ANC visit (no visit)	-19	95	80	1:02	67.00%	27.00%	2.4	22	44	66
8.Poor maternal knowledge in child immunization	-19	95	80	1:02	38.20%	11.20%	4.1	33	66	99

### Sampling procedures

We randomly selected 11 kebeles using the lottery method from the 22 kebeles in the district. All eligible cases and controls in the 11 kebeles were listed from the health post EPI registration books. A house-to-house survey was then conducted to confirm the eligibility of the children identified from the registration books. Children who had permanently changed their residence or did not meet the eligibility criteria of a case or control were excluded from the study. After the survey, a sampling frame was prepared for each kebele, and 90 cases and 180 controls were selected by a simple random sampling technique proportionally to the size of each kebele using computer based Open Epi software.

### Data collection procedures and data quality assurances

A structured questionnaire was adapted from a previous study[20]. The questionnaire was prepared in English and translated into the local language (Tigrigna) and then back translated into English to ensure consistency. The Tigrigna version of the questionnaire was used for data collection. The data were collected through face-to-face interviews with the mothers/caregivers and through a review of the immunization cards. Three supervisors and nine data collectors were trained to recruit the study participants and to administer the questionnaires. All of the data collectors were diploma holders and had experience in collecting data for child health surveys. Training was given for two days to the supervisors and data collectors on how to facilitate the data collection. After the training, a pre-test was conducted among 15 children (9-23months) in two kebeles that were not included in the study. The supervisors checked completeness and consistency of the collected data on daily basis.

### Data processing and analysis

The data were entered into Epi Info version 3.5.1 and analyzed using SPSS version 21. Descriptive statistics were presented using frequencies and graphs. Multicollinearity was tested using variance inflation factor (VIF) and independent variables with VIF > ten were removed. All variables with a p-value ≤ 0.25 in the bivariate analysis were included in a multiple logistic regression model. Hosmer-Leme show goodness-of-fit was used to test the model’s fitness and the value was non-significant (significant level = 0.956), indicating an adequate fit. The model performance was explained between 34.5% (Cox & Snell R square) and 47.9% (Nagelkerke R square) and 80.7% of the children over all were correctly classified by the model.

### Operational definition

Case (Defaulting from completion of immunization): A child between 9–23 months old who had missed at least one dose of the recommended routine vaccination schedule at the time of data collection.

Control (Completion of immunization): A child between 9–23 months old who had received all recommended routine vaccinations at the time of data collection.

Maternal/Caregiver knowledge on child immunization was assessed using eight immunization knowledge related questions. Correct answers were given a score of one and incorrect answers were scored zero. Those scoring greater than the mean were considered to have a satisfactory knowledge and those who scored below the mean were considered to have poor knowledge.

Women's Development Army/Groups (WDA/WDGs) is an organized movement of the community through participatory learning and action meetings. WDGs consist of groups of 25 women residing in a neighborhood in a one-to five networks with one leader and five members.

Maternal participation in the Women Development Groups (WDGs) was assessed using four participation related questions (WDGs had plans regarding child immunization or not; WDGs had performance measurement system or not; one-to-five networks had a weekly meeting or not; and, mothers had own plan regarding child immunization or not). Those mothers/caregivers who were members of a WDG and participated in two or more activities were considered to have satisfactory participation and mothers/caregivers who were not members of a WDG or participated in fewer than two activities were considered as having poor participation.

### Ethical consideration

Verbal informed consent was obtained from each study participant before the interview. Confidentiality was maintained and participants were informed of their right to withdraw from the interview at any stage. Those identified as defaulters were immunized according to EPI. The study protocol was reviewed and approved by the institutional ethical review committee of Mekelle University, College of Health Sciences (Reference number: REC0510/2015).

## Results

### Socio-demographic characteristics of mothers/caregivers

Two hundred and seventy respondents participated in the study, for a response rate of 100%. Of these, 32 mothers of cases (35%), and 44 controls (24.4%) were between the ages of 25–29 years. The mean age of the respondents was 27.93(±6.13) years among cases and 28.04(±3.81) years among controls. The majority, 66 mothers or cases (73.3%) and 112 controls (62.2%) were illiterate. Seventy-eight mothers of cases (86.7%) and 160 controls (88.9%) were currently in WDGs and 85 mothers of cases (94.4%) and 167 controls (92.8%) were housewives. Thirty-six (40%) households of cases and 55 controls (30.6%) had an average family monthly income of <22US Dollar. The remaining demographics are reported in **Table 3.**

**Table 3:** Socio-demographic characteristics of mothers/caregivers on determinants of defaulting from completion of immunization among children aged 9–23 months, in the Laelay Adiabo District, Tigray Region, Northern Ethiopia, 2015 (N = 270).

Variables	Cases, n (%)	Control, n (%)
**Maternal Age (yrs.)**		
<20	10(11.1)	27(15)
20–24	15(16.7)	31(17.2)
25–29	32(35.6)	44(24.4)
30–34	15(16.7)	38(21.1)
35–39	12(13.3)	31(17)
≥40	6(6.7)	9(5)
**Marital status**		
Currently in union	78(86.7)	160(88.9)
Currently not in union**	12(13.3)	20(11.1)
**Place of residence**		
Urban	6(6.7)	28(15.6)
Rural	84(93.3)	152(84.4)
**Maternal educational status**		
Illiterate	66(73.3)	112(62.2)
Literate***	24(26.7)	68(37.8)
**Maternal occupation**		
Housewives	85(94.4)	167(92.8)
Employed*	5(5.6)	13(7.2)
**Husband’s educational level**		
Illiterate	47(52.2)	58(32.2)
Literate***	43(47.8)	112(67.8)
**Husband’s occupation**		
Farmer	82(91.1)	148(82.2)
Employed*	8(8.9)	32(17.8)
**Birth order**		
1st– 3rd	60(54.4)	96(53.3)
4th and above	30(45.6)	84(46.7)
**Family size**		
<5	51(56.7)	106(58.9)
≥5	39(43.3)	74(41.1)
**Monthly family income in US dollar**		
<22USD	36(40)	55(30.6)
22–44 USD	29(32.2)	67(37.2)
>44 USD	25(27.8)	58(32.2)

*Employed (Daily laborer, merchant, government employee)

**Not in Union (single, widowed, divorced)

***literate (Able to read & write, primary, secondary and above).

### Health service related characteristics of mothers /caregivers

This study shows that 70 cases (77.8%) and 162 controls (90%) lived within one hour’s distance from a health facility. Fifty-seven (63.3%) mothers of the cases and 158 controls (87.8%) spent ≤30 minutes to reach vaccination sites. Seventy-six mothers of cases (84.4%) and 170 controls (94.4%) had Antenatal Care (ANC) follow up. Thirty-six (40%) mothers of cases and 110 controls (61.1%) delivered in a health facility. Thirteen mothers of cases (14.4%) and 98 controls (54.4%) had postnatal care (PNC). Fourteen (15.6%) of cases and 12 controls (6.7%) had postponed the vaccine schedule. Twenty-six (28.9%) households of cases and 114 controls (63.3%) were visited by HEWs at least monthly.

### Maternal /caregiver participation in women’s development group (WDG)

The majority, 74 mothers of cases (82.2%) and 152 controls (84.4%) reported being members of a WDG, but only 14 mothers of cases (15.6%) and 89 controls (49.4%) had satisfactory participation in WDGs according to the study criteria. Among the WDG members, 23 mothers of cases (31.1%) and 105 controls (69.1%) had a plan regarding child immunization. Fifteen mothers of cases (20.3%) and 74 controls (48.7%) attended one-to- five union meetings regularly. Two mothers of cases (2.7%) and 20 controls (13.2%) had their own plan about child immunization. Eight mothers of cases (10.8%) and 53 controls (34.9%) had a performance measurement system in the WDGs.

### Knowledge of mothers /caregivers on/about child immunization

Of the total respondents, 84 mothers of cases (93.3%) and 172 controls (95.6%) reported that they had heard about childhood vaccination and vaccine preventable diseases, and 31 mothers of the cases (34.4%) and 93 controls (51.7%) could name five or more types of vaccine preventable diseases. Almost all mothers of cases (98.9%) and controls (99.4%) knew the local vaccination dates, but only 38 mothers of cases (42.2%) and 105 controls (58.3%) knew the child vaccination schedules.

Twenty-nine mothers of cases (32.2%) and 104 controls (57.8%) knew when a child should begin immunization. Most 71 mothers of cases (78.9%) and 174 controls (96.7%) knew when to return for second/third vaccinations. Eighty-two mothers of cases (91.1%) and 179 controls (99.4%) were acquainted with the age when a child should complete immunization. Thirty-two mothers of cases (35.6%) and 111 controls (61.7%) were acquainted with the total number of sessions to complete child immunization. Out of the total, 23 mothers of cases (25.6%) and 127 controls (70.6%) scored above the mean score of 5.83 (±1.22 SD) and were classified as having satisfactory knowledge on child immunization.

### Reasons for defaulting from completion of child immunization

**Fig 1:**
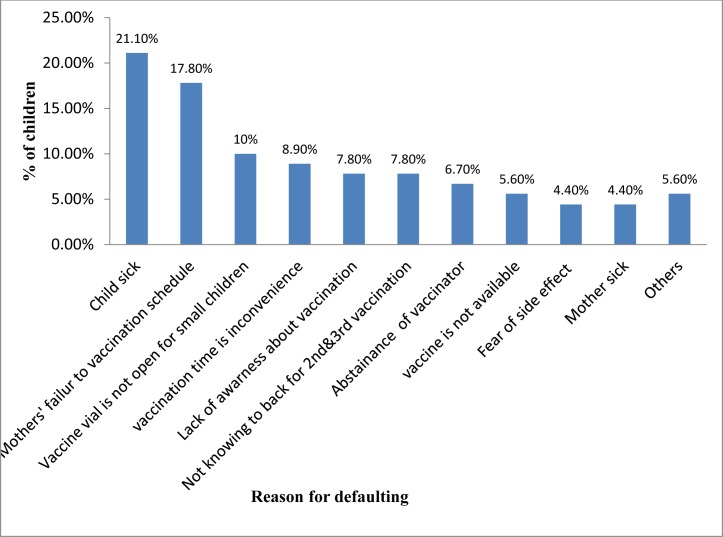
Reasons for defaulting from completion of child immunization among respondents in the Laelay Adiabo District, Tigray, Northern Ethiopia, 2015 (N = 90).

The main reasons for immunization defaulting were child sickness at the scheduled time, reported in 19 of 90 cases (21.1%), mother's failure to attended vaccination schedules (forgetfulness, going to other places or social activities during scheduled vaccinations), reported in 16 of 90 cases (17.8%), and vaccine vials not being opened for a small number of children reported in 9 out of 90 cases (10%)) (**Fig 1)**.

### Factors associated with defaulting from completion of child immunization

Thirteen variables had a p-value≤0.25 in bivariate analysis and were entered into the multivariable logistic regression model. Five of them were independent predictors of defaulting from completion of child immunization after adjusting for all the other variables. Children born from mothers who had no PNC follow-up had five times more likely the odds of defaulting than children born from mothers who had PNC follow-up (AOR = 5.20,95%CI:2.36–11.46). Children living >30 minutes from the vaccination site (AOR = 3.56,95%CI:1.58–8.01), households not visited by HEWs at least monthly (AOR = 2.68,95%CI:1.30–5.51), poor maternal participation in women’s development groups (AOR = 3.3,95%CI:1.54–7.08) and poor knowledge on child immunization (AOR = 3.73,95%CI:1.87–7.43) were significantly associated with defaulting, at a significance level of p-value ≤0.05 (**Table 4**).

**Table 4:** Multivariable logistic regression analysis of selected variables on defaulting from completion of immunization among children aged 9–23 months in the Laelay Adiabo District, Tigray Region, Ethiopia, 2015 (N = 270).

	Defaulting from completion of immunization			
Variables	Cases (%)	Controls (%)	COR(95% CI)	AOR(95% CI)
**Place of residence**				
Urban	6(6.7%)	28(15.6%)	1	1
Rural	84(93.3%	152(84.4%)	2.58(1.03–6.48)*	0.41(0.10–1.63)
**Maternal educational status**				
Illiterate	66(73.3%)	112(62.2%)	1.67(0.96–2.91)	1.1(0.50–2.42)
Literate	24(26.7%)	68(37.8%)	1	1
**Husband’s educational level**				
Illiterate	47(52.2%)	58(32.2%)	2.3(1.37–3.86)*	1.2(0.59–2.44)
Literate	43(47.8%)	122(67.8%)	1	1
**Husband’s occupation**				
Farmer	82(91.1%)	148(82.2%)	2.22(0.98–5.03)	1.1(0.33–3.75)
Employed	8(8.9%)	32(17.8%)	1	1
**Time taken to reach health facility**				
≤ 60 minutes	70(77.8%)	162(90.0%)	1	1
>60 minutes	20(22.2%)	18(10.0%)	**2.57(1.28–5.16**)*	1.11(0.43–2.84)
**Time taken to reach vaccination site**				
≤ 30 minutes	57(63.3%)	158(87.8%)	1	1
>30minutes	33(36.7%)	22(12.2%)	4.15(2.24–7.72)**	3.56(1.58–8.01)*
**ANC follow-up**				
Yes	76(84.4%)	170(94.4%)	1	1
No	14(15.6%)	10(5.6%)	3.13(1.33–7.37)*	0.92(0.30–2.82)
**Place of delivery**				
Home	54(60.0%)	70(38.9%)	2.36(1.41–3.95)*	1(0.49–2.02)
Health Institution	36(40.0%)	110(61.1%)	1	1
**PNC follow-up**				
Yes	13(14.4%)	98(54.4%)	1	1
No	77(85.6%)	82(45.6%)	7.1(3.67–13.65)**	5.2(2.36–11.46)**
**Postponed vaccine schedule**				
Yes	14(15.6%)	12(6.7%)	2.58(1.14–5.84)*	2.7(0.80–9.05)
No	76(84.4%)	168(93.3%)	1	1
**Household visits by HEW at least monthly**				
Yes	26(28.9%)	114(63.3%)	1	1
No	64(71.1%)	66(36.7%)	4.25(2.46–7.35)**	2.68(1.30–5.51)*
**Maternal participation in WDG**				
Satisfactory	14(15.6%)	89(49.4%)	1	1
Poor	76(84.4%)	91(50.6%)	5.3(2.80–10.06)**	3.3(1.54–7.08)*
**Maternal knowledge on Child Immunization**				
Satisfactory	23(25.6%)	127(70.6%)	1	1
Poor	67(74.4%)	53(29.4%)	6.98(3.94–12.37)**	**3.73(1.87–7.43)**

*statistically significant at 0.05<p<0.001

** statistically significant at p<0.001

**model fitness =** 0.956

## Discussion

This study shows that time taken to reach vaccination sites, PNC follow-up, household visits by HEWs at least monthly, maternal participation in WDGs and maternal knowledge of child immunization were predictors of defaulting from completion of child immunization after adjusting for all the other variables.

Time taken to reach the vaccination site was an independent predictor of defaulting from completion of child immunization. This is consistent with a study done in Sudan [21]. Studies in Cameroon and Pakistan also found that long distance to vaccination site a predictor of incomplete child immunization and DTP3 coverage [22, 23]. This may be because the time spent reaching the vaccination site presents a high opportunity cost to mothers/caregivers, especially when vaccine vials were not opened for a small number of children, thus creating the need for multiple visits. This could force mothers to default their children from completion of immunization.

This study revealed that lack of PNC follow up was strongly associated with defaulting from the recommended childhood immunization schedule. Along with other studies from Ethiopia and Kenya with similar findings [18, 24, 25], this implies that mothers are getting adequate information about the child vaccination schedule during their PNC visits.

In this study, children from households who were not receiving a monthly visit from a HEW were more likely to default on immunization. This supports findings from Oromia and Somali regions of Ethiopia [13, 26]. This implies that HEW household visits are an important medium for delivery of health education about child immunization and other health care services. HEW household visits could also provide an opportunity to trace defaulter children in the household.

The likelihood of defaulting from completion of child immunization was more likely higher among mothers who have poor WDG participation. Promoting immunization through community participation is a proven means to build trust and acceptance of child vaccination [27]. Women’s participation in health care decision-making enables women to decide independently to have their child fully vaccinated [28]. This finding implies that the WDGs have become effective in increasing awareness about childhood immunization and in reducing the dropout rate by tracing immunization defaulters in the communities.

Mothers who have poor knowledge about childhood immunization were four times more likely to default from completion of child immunization than those of mothers with satisfactory knowledge. This is consistent with a study conducted in a different part of Ethiopia and a study from Nigeria that both showed that maternal knowledge about child immunization has an independent and significant association with completion of child immunization [13, 15, 17, 18, 19, 29]. Lack of access to information and knowledge about, by whom, where, and when children should be vaccinated were significantly associated with defaulting from completion of children vaccinated [30, 31]. This similarity might be if a mother lacks knowledge on child immunization when her child starts immunization, returns to second/third immunization and completes immunization, may default her child from completing immunization. This finding implies that it is crucially important to increase mother’s awareness about immunization through locally appropriate mechanisms such as HEW visits, WDGs, and PNC. Perhaps the content of immunization health education should be reviewed.

### Limitations of the study

Study participants had difficulty remembering the age and vaccination dose of the child. Additionally, over-reporting opinions and behaviours that are congruent with values deemed socially acceptable and under-reporting those deemed socially undesirable might have been introduced in this study.

## Conclusion and recommendation

The study identified that lack of PNC follow-up, poor maternal knowledge on child immunization, longer time to reach vaccination sites, lower maternal participation in WDGs, and infrequent household visits by HEWs were significant predictors of defaulting from completion of child immunization. PNC visits, HEW household visits, and WDG meetings are important mediums for disseminating information to mothers/caregivers. Therefore, to reduce the rate of defaulters, health providers should motivate and counsel mothers to attend PNC at the health facility. Home based PNC follow-up should be strengthened to improve access for those mothers unable to attend the health facility based PNC follow-up. Mother’s participation in WDGs should be strengthened, and evidence based information on child immunization schedules should be disseminated during the monthly discussions, including the benefits and schedules of routine immunization. HEWs should visit households at least once per month to provide skill based health education and trace defaulters. This may require improved supervision to ensure that household visits are conducted on schedule. Program managers and district health offices should investigate the distribution of vaccination sites and add new sites as needed to address the issue of long travel times. Further studies are needed on effective tracing mechanisms.

## Abbreviations

ANC: Antenatal Care
AOR: Adjusted Odds Ratio
BCG: Bacillus Calmette-Guerin
CI: Confidence Interval
COR: Crude Odds Ratio
DPT: Diphtheria, Pertussis and Tetanus
DPT-HepB-Hib: Diphtheria, Pertussis, Tetanus, Hepatitis B and Haemophilus influenza type B
EPI: Expanded Program on Immunization
HEW: Health Extension Worker
OPV: Oral Polio Vaccine
PCV: Pneumococcal Conjugated Vaccine
PNC: Postnatal Care
SPSS: Statistical Package for Social Sciences
WDG: Women’s Development Group
WHO: World Health Organization

## References

[1] WHO, UNICEF. Global Immunization Vision and Strategy 2006–2015. Geneva: WHO, Department of Immunization, Vaccines and Biologicals; 2005.

[2] Federal Ministry of Health. Immunization in practice national guideline for health workers. 2014.

[3] Federal Ministry of Health. Ethiopia national expanded program on immunization, comprehensive multi-year plan 2011–2015. Addis Ababa; 2010.

[4] Waisbord S, Larson H. Why Invest in Communication for Immunization: Evidence and Lessons Learned. Baltimore, New York: A joint publication of the Health Communication Partnership based at the Johns Hopkins Bloomberg School of Public Health and the United Nations Children’s Fund; 2005.

[5] Vakili R, Hashemi AG, Khademi G, Abbasi MA, Saeidi M. Immunization Coverage in WHO Regions: A Review Article. Int J Pediatr 2015;3 (2–1): 111–8.

[6] WHO, UNICEF, World Bank. State of the world’s vaccines and immunization 3rd ed. Geneva: World Health Organization; 2009.

[7] Federal Ministry of Health. Vaccine management training manual for EPI coordinators and focal persons. 2013.

[8] Federal Demographic Republic of Ethiopia Ministry of Health. Ethiopia Routine Immunization Improvement Plan. 2013.

[9] Federal Democratic Republic of Ethiopia Ministry of health. Policy guidelines on the national expanded program in Ethiopia. Addis Abeba, 2011.

[10] Federal Democratic Republic of Ethiopia Ministry of Health. Policy and practice information for action. Quarterly Health Bulletin 2014;6(1).

[11] Tekile H, Bekele A, Tadesse M, Getachew T, Patterson J, Grant G, et al Ethiopian national immunization coverage survey. Ye science ADMAS 2013 (3).

[12] Maina LC, Karanja S, Kombich J. Immunization coverage and its determinants among children aged 12–23 months in a peri-urban area of Kenya. Pan African Medical Journal 2013;14:3 doi: 10.11604/pamj.2013.14.3.2181 23504493 10.11604/pamj.2013.14.3.2181 PMC3597865

[13] Mohammed H, Atomsa A. Assessment of Child Immunization Coverage and Associated Factors in Oromia Regional State, Eastern Ethiopia. Sci technol arts Res J 2013;2(1):36–41.

[14] Awasthi A, Pandey C, Singh U, Kumar S, Singh T. Maternal Determinants of Immunization status of Children aged 12–23 months in urban slums of Varanasi, India. Clinical Epidemiology and Global Health 2014(1–7).

[15] Debie A, Taye B. Assessment of fully vaccination coverage and associated factors among children aged 12–23 months in Mecha District, North West Ethiopia: A cross-sectional study. Science Journal of Public Health 2014;2(4):342–8.

[16] Sanou A, Simboro S, Kouyate B, Dugas M, Graham J, Bibeau G. Assessment of factors associated with complete immunization coverage in children aged 12–23 months: a cross-sectional study in Nouna District, Burkina Faso. BMC International Health and Human Rights 2009;9(Suppl 1):10. 19828054 10.1186/1472-698X-9-S1-S10 PMC2762310

[17] Odusanya O, Alufohai E, Meurice F, Ahonkhai V. Determinants of vaccination coverage in rural Nigeria. BMC Public Health 2008;8:381 doi: 10.1186/1471-2458-8-381 18986544 10.1186/1471-2458-8-381 PMC2587468

[18] Tadesse H, Deribew A, Woldie M. Predictors of defaulting from completion of child immunization in south Ethiopia, May 2008 –A case control study. BMC Public Health 2009;9:150 doi: 10.1186/1471-2458-9-150 19463164 10.1186/1471-2458-9-150 PMC2694177

[19] Tesfaye F, Tamiso A, Birhan Y, Tadele T. Predictors of Immunization Defaulting among Children Age 12–23 Months in Hawassa Zuria District of Southern Ethiopia. International Journal of Public Health Science 2014;3(3):185–93.

[20] Etana B, Deressa W. Factors associated with complete immunization coverage in children aged 12–23 months in Ambo Woreda, Central Ethiopia. BMC Public Health 2012;12:566 doi: 10.1186/1471-2458-12-566 22839418 10.1186/1471-2458-12-566 PMC3508824

[21] Ibnouf A. Factors influencing immunization coverage among children under five years of age in Khartoum State, Sudan. SA Fam Pract 2007;49(8):14.

[22] Russo G, Miglietta A, Pezzotti P, Biguioh RM, Mayaka GB, Sobze MS, et al Vaccine coverage and determinants of incomplete vaccination in children aged 12–23 months in Dschang, West Region, Cameroon: a cross-sectional survey during a polio outbreak. BMC Public Health 2015;15: 630 doi: 10.1186/s12889-015-2000-2 26156158 10.1186/s12889-015-2000-2 PMC4496879

[23] Usman H, Kristensen S, Rahbar H, Vermund S, Habib F, Chamot E. Determinants of third dose of diphtheria–tetanus–pertussis (DTP) completion among children who received DTP1 at rural immunization centres in Pakistan: a cohort study. Tropical Medicine and International Health 2010;15(1):140–7. doi: 10.1111/j.1365-3156.2009.02432.x 19930140 10.1111/j.1365-3156.2009.02432.x PMC2858790

[24] Yenit MK, Assegid S, Abrha H. Factors Associated With Incomplete Childhood Vaccination among Children 12–23 Months of Age in Machakel Woreda, East Gojjam Zone: A Case Control Study Journal of Pregnancy and Child Health 2015;2:4.

[25] Mutua MK, Kimani-Murage E, Ngomi N, Ravn H, Mwaniki P, Echoka E. Fully immunized child: coverage, timing and sequencing of routine immunization in an urban poor settlement in Nairobi, Kenya. Tropical Medicine and Health 2016;44:13 doi: 10.1186/s41182-016-0013-x 27433132 10.1186/s41182-016-0013-x PMC4940963

[26] Mohamud AN, Feleke A, Worku W, Kifle M, Sharma HR. Immunization coverage of 12–23 months old children and associated factors in Jigjiga District, Somali National Regional State, Ethiopia. BMC Public Health 2014;14:865 doi: 10.1186/1471-2458-14-865 25146502 10.1186/1471-2458-14-865 PMC4158082

[27] Kidane T, Yigzaw A, Sahilemariam Y, Bulto T, Mengistu H, Belay T, et al National EPI coverage survey report in Ethiopia. Ethiop J Health Dev 2008;22(2):148–57.

[28] Wado Y, Afework M, Hindin I. Childhood vaccination in rural southwestern Ethiopia: the nexus with demographic factors and women’s autonomy. Pan African Medical Journal 2014;17(Supp 1):9. 10.11694/pamj.supp.2014.17.1.3135 PMC3946289 24624243

[29] Legesse E, Dechasa W. Assessment of child immunization coverage and its determinants in Sinana District, Southeast Ethiopia. BMC Pediatrics. 2015;15:31 doi: 10.1186/s12887-015-0345-4 25886255 10.1186/s12887-015-0345-4 PMC4438454

[30] Siddiqabugvi A, Rahat R, Zakar R, Zakriazakar M, Fischer F, Nasrullah M, et al Factors associated with non-utilization of child immunization in Pakistan: evidence from the Demographic and Health Survey 2006–07. BMC Public Health 2014;14:232 doi: 10.1186/1471-2458-14-232 24602264 10.1186/1471-2458-14-232 PMC3973983

[31] Dube E, Laberge C, Guay M, Bramadat P, Roy R, Bettinger J. Vaccine hesitancy an overview. Human Vaccines & Immunotherapeutics 2013;9(8):1763–73. 23584253 10.4161/hv.24657 PMC3906279

